# Morris Water Maze Experiment

**DOI:** 10.3791/897

**Published:** 2008-09-24

**Authors:** Joseph Nunez

**Affiliations:** Department of Psychology, Michigan State University

## Abstract

The Morris water maze is widely used to study spatial memory and learning. Animals are placed in a pool of water that is colored opaque with powdered non-fat milk or non-toxic tempera paint, where they must swim to a hidden escape platform. Because they are in opaque water, the animals cannot see the platform, and cannot rely on scent to find the escape route. Instead, they must rely on external/extra-maze cues.  As the animals become more familiar with the task, they are able to find the platform more quickly. Developed by Richard G. Morris in 1984, this paradigm has become one of the "gold standards" of behavioral neuroscience.

**Figure:**
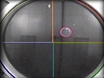


## Protocol

### Setting up the water maze

The main component of the water maze set up should be a round pool, about 6 feet in diameter and about 3 feet deep. If you are recording the task with a video camera, make sure all sides of the maze are within the camera’s field of view.
Fill up the water maze with tap water, which should be close to 26°C. This may take several hours, so should be done well in advance. Periodically check the water temperature so that it is within one degree of 26°C.
Place the escape platform in the center of the pool. During training, it must be exposed, one inch above the water. This teaches the rat that there is a platform, and that it is the way to get out of the water. Later, after the animal is trained and ready for testing, the escape platform will be just below the surface of the water, and will not be visible because the water will be made opaque with milk or non-toxic paint.    Now, the water maze is ready for training the animals.

### Pre-training for the water maze

For the water maze training, the platform should be in the center of the pool and exposed one inch above the surface, so the animal knows that it’s there. The water should be within one degree of 26°C.
Each animal will undergo three consecutive trials. First, place the animal on the platform for twenty seconds.
The water maze has 4 starting positions: north, south, east, or west.  Take the animal to one of these positions.  Lower the animal into the water by supporting it with your hand and bringing it down gently into the water tail-end first. Do not stress the animal out by dunking it in head first.
Let the animal swim/search for the platform for a maximum of 60 seconds. At first, the animal may swim around the edge of the pool looking for a way out.  Eventually, the animal will learn to search for the platform and climb up. 
Once the rodent reaches the platform, stop the timer, and record the time.  If it doesn’t find the platform in 60 seconds, then record the time for this trial as one minute. Do not pick up the animal if it fails to reach the platform. Teach the animal that it must swim to the platform. Therefore, gently guide the animal to the platform with your hand.  Let the animal sit on the platform for 15 seconds. If it falls or jumps off, gently guide it back. This will train the animal that it must stay on the platform to be rescued from the pool.
Repeat the same procedure for two more trials, starting at a different direction for each trial.
Once the animal has completed all three trials, dry it off with a towel. Repeat the three-trial training process for all the animals consecutively. Keep the directions the same for all of them, and record their times.
Now that the animals are trained, they are ready to perform the water maze test.

### Water maze testing

To begin experimental trials with the water maze, fill up the tank so the platform is one inch below the surface of the water. Use non-fat dry milk, or 125 milliliters of non-toxic white tempera paint, to make the water opaque. 
The lighting and water temperature should be the same as in the training process. Each animal will undergo 12 trials, which will be 3 trials for each starting direction. Each trial will last 60 seconds. Before beginning, choose the order of the starting directions. Do not use the same start direction twice in a row, and also do not repeat the same order for any of the directions
Facing the wall of the pool, the animal handler will place the animal in the water, and will then step back from the pool and sit in a designated spot while the animal performs the maze task. An animal behavior tracking system such as the SMART system (San Diego Instruments) can be used to monitor the path, as well as other variables. Videotaping each trial is a less expensive, and perfectly appropriate, alternative.
Monitor the animal until it reaches the platform, and record the time it took. If the animal doesn’t reach the platform in 60 seconds, the handler will guide it to the platform, as in training. Either way, let the animal sit for 10 seconds, and then dry it off and return the animal to a holding cage.
Continue with each animal for all 12 trials, with the animal handler returning to the same designated spot during each trial. The order of testing should be: trial 1 for all animals, trial 2 for all animals, trial 3 for all animals, etc. There should be an inter-trial interval of at least 2 minutes. Periodically, clean out the pool, make sure the platform is in place, and check that the water temperature is the same.
After all animals have completed 12 trials, they will each perform one probe trial, in which the platform is removed from the pool. The probe trial is performed to verify the animal’s understanding of the platform location, and observe the strategy that the animal follows when it discovers the platform is not there. The handler will release the animal starting from the north. Record the number of times the animal crosses the center of the pool during the  30 seconds.
When all the probe trials are complete, dry off the animals and drain the pool.

## Discussion

The water maze task was development by Morris ^5^. This task can be altered in numerous ways to investigate working memory, reference memory and task strategy ^6^. The procedure described here contains two critical variables which represent a deviation from other versions of the water maze: pretraining, and testing on a single day.

**Pretraining** - the hippocampus is involved in spatial/relational memory ^7-8^. The water maze specifically tests spatial memory ^3^. However, there are numerous other components of the task that do not involve spatial memory: the stress involved with the task, the understanding of the rules of the task (that to "escape", the animal must find a hidden platform, and stay on it in order to be "rescued"), and the understanding that there is a means of escaping the task ^9^. Learned helplessness also involves a tank of water, but the rules (there is no means of escape) are quite different ^10^. The three pretraining trials "teach" the animals about these properties of the task. They learn that they will be placed into a pool of tepid water and swim around for a minute, but be removed after. They are taught to find the platform (because it is visible) and that staying on it will lead to their "escape" from the maze. And they are taught that the task has an end. Therefore, this hippocampal-independent learning does not confound the analysis of the water maze testing data.

**Testing on a single day** - Most often, water maze testing occurs across two to four days ^3^. In this way, acquisition and retention can be assessed. However, in some populations, this is not a viable option. Such is the case when investigating female mammals. Female rodents, humans, primates, etc, all have cyclic changes in steroid hormone levels ^11-12^. These hormones have profound effects on hippocampal-dependent task performance, hippocampal anatomy and hippocampal cell function ^13-14^. Testing across multiple days would be akin to testing a single animal across numerous conditions low estradiol and progesterone, elevated estradiol and progesterone, and intermediate estradiol and progesterone. To eliminate this confound, testing occurs across a single day.
